# Quality of interventional animal experiments in Chinese journals: compliance with ARRIVE guidelines

**DOI:** 10.1186/s12917-020-02664-1

**Published:** 2020-11-26

**Authors:** Bing Zhao, Yanbiao Jiang, Ting Zhang, Zhizhong Shang, Weiyi Zhang, Kaiyan Hu, Fei Chen, Fan Mei, Qianqian Gao, Li Zhao, Joey S. W. Kwong, Bin Ma

**Affiliations:** 1 Evidence Based Medicine Center, School of Basic Medical Sciences, Lanzhou University, No.199, Donggang West Road, Lanzhou City, 730000 Gansu Province China; 2 Second clinical medical college, Lanzhou University, Lanzhou, 730000 China; 3 School of Public Health, Lanzhou University, Lanzhou, 730000 China; 4 School of Nursing, Lanzhou University, Lanzhou, 730000 China; 5 School of Public Health and Primary Medical Care, Jockey Club, Chinese University of Hong Kong, Hong Kong, 999077 China

**Keywords:** Animal experiments, ARRIVE, Reporting quality

## Abstract

**Background:**

In view of the inadequacy and incompleteness of currently-reported animal experiments and their overall poor quality, we retrospectively evaluated the reporting quality of animal experiments published in Chinese journals adhering to the Animal Research: Reporting of In Vivo Experiments (ARRIVE) guidelines.

**Results:**

The databases CNKI, WanFang, VIP, and CBM were searched from inception until July 2018. Two appropriately-trained reviewers screened and extracted articles independently. The ARRIVE guidelines were used to assess the quality of the published reports of animal experiments. The compliance rate of every item was analyzed relative to their date of publication. A total of 4342 studies were included, of which 73.0% had been cited ≤5 times. Only 29.0% (1261/4342) were published in journals listed in the Chinese Science Citation Database. The results indicate that the compliance rate of approximately half of the sub-items (51.3%, 20/39) was less than 50%, of which 65.0% (13/20) was even less than 10%.

**Conclusions:**

The reporting quality of animal experiments in Chinese journals is not at a high level. Following publication of the ARRIVE guidelines in 2010, the compliance rate of the majority of its requirements has improved to some extent. However, less attention has been paid to the ethics and welfare of experimental animals, and a number of specific items in the Methods, Results, and Discussion sections continue to not be reported in sufficient detail. Therefore, it is necessary to popularize the ARRIVE guidelines, advocate researchers to adhere to them in the future, and in particular promote the use of the guidelines in specialized journals in order that the design, implementation, and reporting of animal experiments is promoted, to ultimately improve their quality.

**Supplementary Information:**

The online version contains supplementary material available at 10.1186/s12917-020-02664-1.

## Background

Animal experimentation in scientific research is required for the acquisition of new knowledge of mechanisms in biology and medicine, or to answer specific scientific questions [1], and so represents a basic tenet of biomedical research. Animal experiments are also an important aspect of preclinical research. As a bridge between basic research and clinical trials, the quality of animal experiments affects the achievements and conclusions of research studies in many fields [2, 3].

Over recent years, the number of animal experiments published in biomedical journals has increased dramatically. Increasing numbers of studies have shown that [4–7], even in animal experiments published in top journals, the quality of reporting remains unsatisfactory. A review by The National Centre for the Replacement, Refinement and Reduction of Animals in Research, the NC3Rs [8], demonstrated that many funded and published animal experiments had inadequate reporting of important information such as study design, implementation, and analysis. In 271 animal experiments included in the review, 41% did not state the hypothesis or objective of the study or the number and basic characteristics of the animals used in the experiments, and 30% of them did not describe the statistical methods or did not present the results with correct statistical indicators. In addition, good animal welfare is closely related to the reliability and repeatability of animal experiments. Seeking new methods to better promote animal welfare is also a concern of the 3Rs center [9]. The inadequacy and incompleteness of the quality of articles and poor quality of reports have seriously hindered the utilization of the scientific and practical value of those animal experiments. Therefore, in order to improve the quality of animal experimental reports, based on the CONSORT Statement [10], the NC3Rs developed the ARRIVE (Animal Research: Reporting of In Vivo Experiments) guidelines, which were published in 2010. The guidelines consist of six parts: title, abstract, introduction, methods, results, and discussion, with 20 items and sub-items [11]. Since 2010, researchers outside China have studied the quality of animal experimental reports published in published in international journals based on the ARRIVE guidelines [12–14], whereas in China, similar research has only been published by Liu et al [15] Furthermore, their research is limited to specific diseases. In addition, no studies have explored whether publication of the ARRIVE guidelines has improved the quality of animal experimental reports.

Therefore, we collected comprehensively animal experiments published in China, reviewed and analyzed the quality of the experimental reports and existing problems based on the ARRIVE guidelines, in order to provide a reference for the promotion of quality of animal experimental reports in China.

## Results

### Selection process and results

**Fig. 1:**
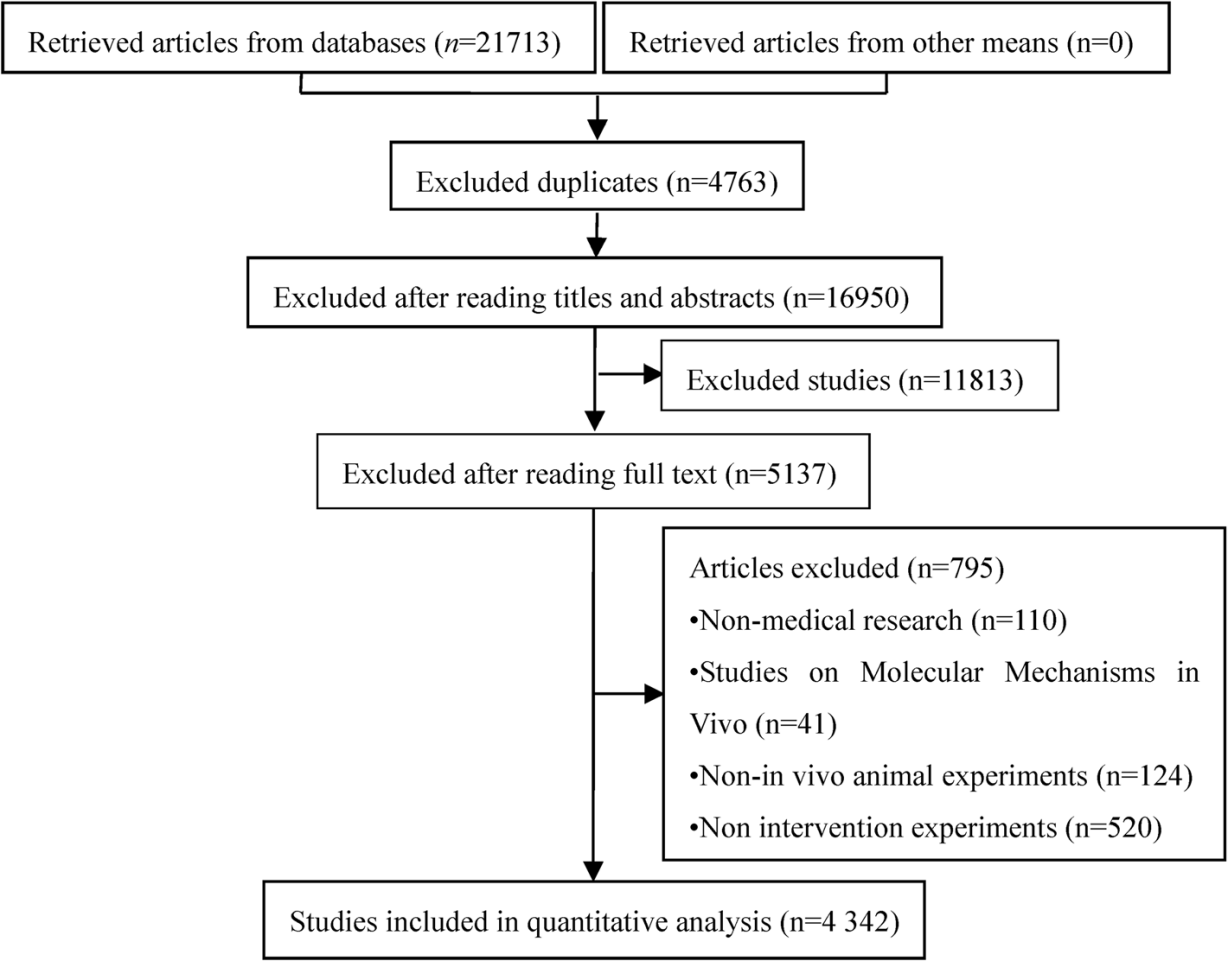
Flow chart for the process of article selection and results. *Retrieved databases and selected studies as follows: CNKI (*n* = 1213), WanFang (*n* = 5500), VIP (*n* = 1702), CBM (*n* = 13,298)

A total of 21,713 potentially relevant studies were initially selected. After the exclusion of duplicates and those that did not fulfill the inclusion criteria, 4342 studies were ultimately included, in which 4925 animal experiments were conducted (as some included multiple animal experiments). The selection process and results are shown in Fig. 1. Each sub-item in each study was deemed either “yes” (total or partial compliance) or “no” (noncompliance), where the former was defined as a study reporting all or a part of at least one information sub-category items in detail. The compliance rate for the 39 sub-items deemed “yes” was calculated (See “Methods” for details).

### Basic characteristics of the studies included in the review

The number of citations for each included study ranged from 0 to 12. More than 70% (73.0%, 3171/4342) had been cited fewer than 5 times, of which nearly 50% (47.6%, 1510/3171) had not been cited at all. Only 29.0% (1261/4342) had been published in journals included in the Chinese Science Citation Database(CSCD), which is a collection of outstanding Chinese and English journals in various fields in China and known as “Chinese SCI”. The greatest proportion of first authors were clinicians (45.1%, 1957/4342) (Table 1).

**Table 1:** Basic characteristics of studies included in the review

Category	Value	Total N (%)
Citations	0	1510 (34.8)
	1—5	1661 (38.3)
	>5	1171 (27.0)
Categories of journals	Professional journals	1991 (45.9)
	Comprehensive journals	1654 (38.1)
	Others	697 (16.1)
Identities of the first author	Clinical doctors	1957 (45.1)
	Postgraduate students	740 (17.0)
	Researchers	1645 (37.9)

**Fig. 2:**
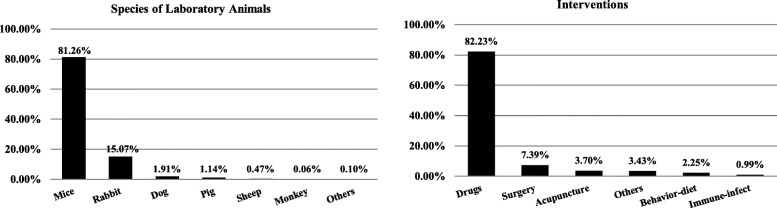
Laboratory animal species and interventions of animal test reports published in Chinese journals

Of the 4925 animal experiments included in the review, the most frequently-used species of animal was the mice (81.3%, 4002/4925), then rabbits (15.1%, 742/4925), and dogs (1.91%, 94/4925). Of the type of intervention, the top three were drugs (82.2%, 4050/4925), surgery (7.39%, 364/4925), and acupuncture (3.70%, 182/4925) (Fig. 2). In coverage and classification of related diseases, the top three were circulatory diseases (10.9%, 538/4925), digestive diseases (10.6%, 524/4925), and tumors (9.66%, 476/4925).

### Reporting quality of studies included in the review (Fig. 3, Table 2)

The ARRIVE guidelines have 6 sections with 20 items and 39 sub-items. Only 28.2% (11/39) of the sub-items were more than 90% compliant. The “yes” compliance rate of approximately half of the sub-items (51.3%, 20/39) was less than 50%, of these “yes” poorly compliant items, 90% (18/20) were less than 30% compliant, while 65% (13/20) were less than 10% compliant.

- ① Items relating to the title, abstract, and introduction.

**Fig. 3:**
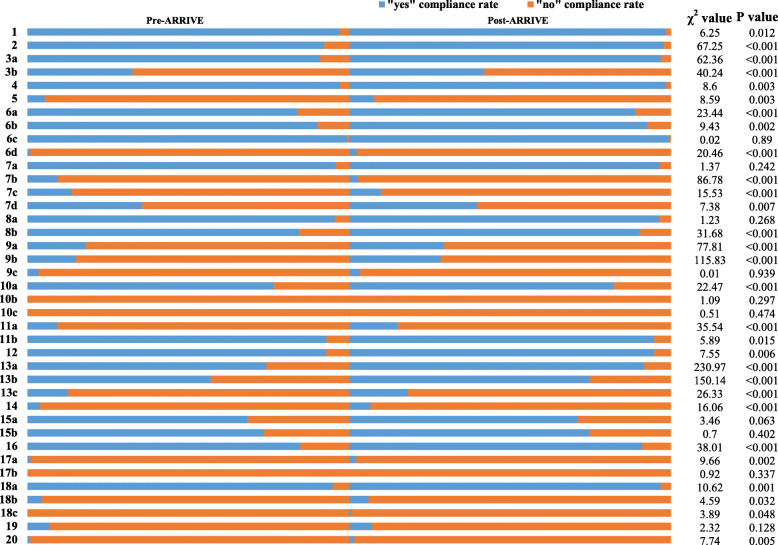
Assessment of reporting quality of included studies

**Table 2:** Assessment of reporting quality of included studies

Content and Subject	Item	Description	Total		pre-ARRIVE		post-ARRIVE		χ^**2**^ value	***P*** value
			items assessed as “yes”		items assessed as “yes”		items assessed as “yes”			
			N	%	N	%	N	%		
**TITLE**										
	1	Provide as accurate and concise a description of the content of the article as possible.	4241	97.7	2196	97.1	2045	98.3	6.25	0.012
**ABSTRACT**										
	2	Provide an accurate summary of the background, research objectives (including details of the species or strain of animal used), key methods, principal findings, and conclusions of the study	4117	94.8	2084	92.2	2033	97.7	67.25	< 0.001
**INTRODUCTION**										
Background	3a	Include sufficient scientific background (including relevant references to previous work) to understand the motivation and context for the study, and explain the experimental approach and rationale.	4071	93.8	2057	91.0	2014	96.8	62.36	< 0.001
	3b	Explain how and why the animal species and model being used can address the scientific objectives and, where appropriate, the study’s relevance to human biology.	1609	37.1	737	32.6	872	41.9	40.24	< 0.001
Objectives	4	Clearly describe the primary and any secondary objectives of the study, or specific hypotheses being tested.	4248	97.8	2198	97.2	2050	98.5	8.60	0.003
**METHODS**										
Ethical statement	5	Indicate the nature of the ethical review permissions, relevant licenses (e.g. Animal [Scientific Procedures] Act 1986), and national or institutional guidelines for the care and use of animals, that cover the research.	286	6.59	125	5.53	161	7.74	8.59	0.003
Study design	6a	For each experiment, give brief details of the study design, including: the number of experimental and control groups.	3741	86.2	1893	83.7	1848	88.8	23.44	< 0.001
	6b	For each experiment, give brief details of the study design, including: any steps taken to minimise the effects of subjective bias when allocating animals to treatment (e.g., randomisation procedure) and when assessing results (e.g., if done, describe who was blinded and when).	3961	91.2	2034	90.0	1927	92.6	9.43	0.002
	6c	For each experiment, give brief details of the study design, including: the experimental unit (e.g. a single animal, group, or cage of animals).	4312	99.3	2245	99.3	2067	99.3	0.02	0.890
	6d	For each experiment, give brief details of the study design, including: A time-line diagram or flow chart can be useful to illustrate how complex study designs were carried out.	63	1.45	15	0.66	48	2.31	20.46	< 0.001
Experimental procedures	7a	For each experiment and each experimental group, including controls, provide precise details of all procedures carried out. For example: How (e.g., drug formulation and dose, site and route of administration, anaesthesia and analgesia used [including monitoring], surgical procedure, method of euthanasia). Provide details of any specialist equipment used, including supplier(s).	4172	96.1	2165	95.8	2007	96.4	1.37	0.242
	7b	For each experiment and each experimental group, including controls, provide precise details of all procedures carried out. For example: When (e.g., time of day).	269	6.20	214	9.46	55	2.64	86.78	< 0.001
	7c	For each experiment and each experimental group, including controls, provide precise details of all procedures carried out. For example: Where (e.g., home cage, laboratory, water maze).	513	11.8	309	13.7	204	9.80	15.53	< 0.001
	7d	For each experiment and each experimental group, including controls, provide precise details of all procedures carried out. For example: Why (e.g., rationale for choice of specific anaesthetic, route of administration, drug dose used).	1631	37.6	806	35.7	825	39.6	7.38	0.007
Experimental animals	8a	Provide details of the animals used, including species, strain, sex, developmental stage (e.g., mean or median age plus age range), and weight (e.g., mean or median weight plus weight range).	4162	95.9	2160	95.5	2002	96.2	1.23	0.268
	8b	Provide further relevant information such as the source of animals, international strain nomenclature, genetic modification status (e.g. knock-out or transgenic), genotype, health/immune status, drug- or testnaive, previous procedures, etc.	3785	87.2	1909	84.4	1876	90.2	31.68	< 0.001
Housing and husbandry	9a	Housing (e.g., type of facility, specific pathogen free (SPF); type of cage or housing; bedding material; number of cage companions; tank shape and material etc. for fish).	1022	23.5	409	1819	613	29.5	77.81	< 0.001
	9b	Husbandry conditions (e.g., breeding programme, light/dark cycle, temperature, quality of water etc. for fish, type of food, access to food and water, environmental enrichment).	928	21.4	338	15.0	590	28.4	115.83	< 0.001
	9c	Welfare-related assessments and interventions that were carried out before, during, or after the experiment.	147	3.39	77	3.41	70	3.36	0.01	0.939
Sample size	10a	Specify the total number of animals used in each experiment and the number of animals in each experimental group.	3440	79.2	1728	76.4	1712	82.3	22.47	< 0.001
	10b	Explain how the number of animals was decided. Provide details of any sample size calculation used.	1	0.02	0	0.00	1	0.05	1.09	0.297
	10c	Indicate the number of independent replications of each experiment, if relevant.	6	0.14	4	0.18	2	0.10	0.51	0.474
Allocating animals to experimental groups	11a	Give full details of how animals were allocated to experimental groups, including randomization or matching if done.	522	12.0	208	9.20	314	15. 1	35.54	< 0.001
	11b	Describe the order in which the animals in the different experimental groups were treated and assessed.	4070	93.7	2100	92.9	1970	94.7	5.89	0.015
Experimental outcomes	12	Clearly define the primary and secondary experimental outcomes assessed (e.g., cell death, molecular markers, behavioral changes).	4071	93.8	2098	92.8	1973	94.8	7.55	0.006
Statistical methods	13a	Provide details of the statistical methods used for each analysis.	3585	82.6	1677	74.2	1908	91.7	230.97	< 0.001
	13b	Specify the unit of analysis for each dataset (e.g. single animal, group of animals, single neuron).	2840	65.4	1287	56.9	1553	74.6	150.14	< 0.001
	13c	Describe any methods used to assess whether the data met the assumptions of the statistical approach.	662	15.3	284	12.6	378	18.2	26.33	< 0.001
**RESULTS**										
Baseline data	14	For each experimental group, report relevant characteristics and health status of animals (e.g., weight, microbiological status, and drug- or test-naı¨ve) before treatment or testing (this information can often be tabulated).	223	5.14	87	3.85	136	6.54	16.06	< 0.001
Numbers analysed	15a	Report the number of animals in each group included in each analysis. Report absolute numbers (e.g.10/20, not 50%)	3023	69.6	1546	68.4	1477	71.0	3.46	0.063
	15b	If any animals or data were not included in the analysis, explain why.	3213	74.0	1661	73.5	1552	74.6	0.70	0.402
Outcomes and estimation	16	Report the results for each analysis carried out, with a measure of precision (e.g, standard error or confidence interval).	3811	87.8	1918	84.8	1893	91.0	38.01	< 0.001
Adverse events	17a	Give details of all important adverse events in each experimental group.	64	1.47	21	0.93	43	2.07	9.66	0.002
	17b	Describe any modifications to the experimental protocols made to reduce adverse events.	1	0.02	1	0.04	0	0.00	0.92	0.337
**DISCUSSION**										
Interpretation/scientific implications	18a	Interpret the results, taking into account the study objectives and hypotheses, current theory, and other relevant studies in the literature.	4157	95.7	2143	94.8	2014	96.8	10.62	0.001
	18b	Comment on the study limitations including any potential sources of bias, any limitations of the animal model, and the imprecision associated with the results.	228	5.25	103	4.56	125	6.01	4.59	0.032
	18c	Describe any implications of your experimental methods or findings for the replacement, refinement, or reduction (the 3Rs) of the use of animals in research.	15	0.35	4	0.18	11	0.53	3.89	0.048
Generalisability/translation	19	Comment on whether, and how, the findings of this study are likely to translate to other species or systems, including any relevance to human biology.	281	6.47	134	5.93	147	7.06	2.32	0.128
Funding	20	List all funding sources (including grant number) and the role of the funder(s) in the study.	47	1.08	15	0.66	32	1.54	7.74	0.005

For items 1–2, involving the title and abstract, the “yes” compliance rate was higher than 90%. The “yes” compliance rate of items 1 and 2 in studies published after 2010 was higher than that of studies published before 2010. The differences between the two groups for both items 1 (*P* = 0.012) and 2 (*P* < 0.001) were statistically significant.

For items 3a-4, which involved the abstract, the “yes” compliance rate was higher than 90%, although the “yes” compliance rate of item 3b was only 37.1%. The “yes” compliance rate of items 3a (P < 0.001), 3b (P < 0.001) and 4 (*P* = 0.003) in studies published after 2010 was higher than that of studies published before 2010, and the differences between the two years of publication were statistically significant, for item.

- ② Items relating to methodology.

For 28 items (items 5-13c) involving methodology, the “yes” rate of compliance for items 6a, 6b, 6c, 7a, 8a, 8b, 10a, 11b, 12, 13a, and 13b was greater than 50%. The “yes” compliance rate of items 6b, 6c, 7a, 8a, 11b, and 12 was greater than 90%, but less than 50% for items 5, 6d, 7b, 7c, 7d, 9a, 9b, 9c, 10b, 10c, 11a, and 13c. Except for item 7d, the “yes” rate of compliance in the latter group was less than 30%, and for items 5, 6d, 7b, 9c, 10b, and sub-item 10c it was even less than 10%. The compliance rate for items 5 (*P* = 0.003), 6a (*P* < 0.001), 6b (*P* = 0.002), 6d (P < 0.001), 7d (*P* = 0.007), 8a (P < 0.001), 9a (P < 0.001), 9b (*P* < 0.001), 10a (*P* < 0.001), 11a (P < 0.001), 11b (*P* < 0.015), 12 (*P* < 0.006), 13a (P < 0.001), 13b (P < 0.001), and 13c (P < 0.001) in studies published after 2010 was slightly higher than those before 2010, but not for 6c (*P* = 0.890), 7a (*P* = 0.242), 8a (*P* = 0.268) or 10b (*P* = 0.297). The differences between the two publication year groups were significantly different for items. However, the “yes” compliance rate for items 7b (*P* < 0.001), 7c (P < 0.001) in studies published after 2010 was even lower than in reports published before 2010 but not for 9c (*P* = 0.939) and 10c (*P* = 0.474).

- ③ Items relating to the results and discussion.

For items 14-17b involving the results, the “yes” compliance rate of items 15a, 15b, and 16 was greater than 50%, while for items 14, 17a, and 17b it was less than 6%. The “yes” compliance rate for items 14 (P < 0.001), and 17a (*P* = 0.002) for studies published after 2010 was higher than in studies published after 2010, but not for items 15a (*P* = 0.063), 15b (*P* = 0.402), or 17b (*P* = 0.337).

For items 18a-20 involving the discussion, except for the “yes” compliance rate of item 18a, which was greater than 90%, that of the other items was lower than 10%. The “yes” compliance rate of the 5 items in studies published after 2010 was higher than those of studies published prior to 2010 for items 18a (*P* = 0.001), 18b (*P* = 0.032), 18c (*P* = 0.048), and 20 (*P* = 0.005), but not for item 19 (*P* = 0.128).

## Discussion

The present study is the first comprehensive and systematic review of the reporting quality of animal experiments published in Chinese journals based on the ARRIVE guidelines. Following publication of the guidelines, the compliance rate of the majority of items improved to a certain extent although there remains continued inadequate reporting of specific items, e.g. study design, procedures, sample size, adverse events, and discussion.

### Methods section

High-quality research is inseparable from scientific design and rigorous implementation. These guidelines (ARRIVE) aimed to maximize the output of animal research by optimizing the information provided in publications regarding experimental design, implementation, and analysis [11]. In the ARRIVE guidelines, methods include nine aspects: ethical statement, study design, experimental procedure, animals, housing and husbandry, sample size, allocation of animals to experimental groups, experimental outcomes, and statistical methods. The present research showed that:

- ① Ethical Statement: Of the animal studies published in Chinese journals, only 6.59% reported an ethical statement (item 5). The compliance rate was slightly higher in studies published after the publication of the ARRIVE guidelines, but only to 7.74%. Nikki Osborne et al. conducted a review of the literature that had published original studies on animals, and the results indicate that a considerable number of journals have not made this a mandatory requirement [16]. A number of studies have shown [17] that at least 20 million animals are used in scientific experiments every year in China, of which approximately 25% suffer pain from the procedure, but approximately 10% are not administered painkillers. An analysis of the ethical status of experimental animals in China by Chen Jie et al. [18] demonstrated these serious problems, where animals are used as a tool to obtain experimental data without considering any suffering they may have to tolerate in the process of experimentation. Highly distressed laboratory animals may lead to unreliable conclusions and/or unnecessary changes in scientific output, thereby affecting the reliability and repeatability of experiments, therefore every effort should be made to reduce unnecessary harm to them [9]. Although all countries have successively introduced laws and policies on animal welfare, and have even established animal protection organizations to supervise the regulations [19, 20], Olsson [21] and other researchers have also discussed how better to promote the issue. However, efforts on animal welfare remain unsatisfactory. Therefore, it is necessary to further regulate animal experimentation through legislation. More attention should be paid to animal ethics, adhering to the “3Rs” principle, and respecting the value and rights of animals.
- ② Study design: After 2010, although more studies used time-line diagrams or flow charts to illustrate the entire process of the design of the study and conduct of the research (item 6d), the actual compliance rate was only 2.31%. A number of studies have shown that more than half of preclinical studies are unrepeatable [22], due to deficiencies in study design, incorrect statistical analysis and inadequate reporting [8, 23, 24]. Therefore, a clear and detailed flow chart can be effective in displaying the entire process of the experiment and improve transparency of the experimental implementation.
- ③ Experimental procedures: Of animal studies published in Chinese journals, only 6.20 and 11.8% of studies provided time of day when experimental procedures take place (item 7b) or where the experiments take place (item 7c), respectively. One study [25] demonstrated that it was not possible to scientifically evaluate the reliability of the results in 76 articles describing animal experimentation (with more than 500 citations) due to a lack of reporting of key information such as experimental process and procedures in the methodological part of the experiments. Detailed reporting of experimental processes and procedures is a key measure to ensure replication of the results is possible and improve their utilization and conversion rate.
- ④ Housing and procedures for husbandry: Although research published after 2010 showed that the compliance rate for housing (item 9a) and husbandry (item 9b) improved (*P* < 0.001), actual compliance rates were only 29.5 and 28.4%, respectively. It is well known that the type of cage or housing, style of settlement, light/dark cycle, temperature, and water quality will impact experimental results. As Hooijmans et al. [2, 26–28] pointed out in their research that there can be a four-fold difference in light intensity between cages at the top and bottom of a rack, and small differences such as these have been associated with animal reproduction and behavior.
- ⑤ Sample size: Of 4342 animal studies published in Chinese journals, only one study reported the algorithm and formula for calculating sample size (item 10b), and only six studies indicated the number of independent replications of each experiment (item 10c). The National Institute of Neurological Disorders and Stroke (NINDS) reached a consensus in 2012 on how to improve reporting in animal research reports for submission of articles and for applying for funding, in which they noted [29] “insufficient sample size may lead to false-negative results, and miss some potentially important discoveries”. Therefore, it is necessary to report the number of animals used in each group and the statistical methods used to calculate sample size.
- ⑥ Allocation of animals to experimental groups: Although the rate of reporting of specific grouping methods for experimental animals published after 2010 (item 11a) was significantly higher than those published before 2010 (*P* < 0.001), the actual coincidence rate was only 12.0%. A number of studies have shown [8] that the process of randomization, allocation concealment, and blinding are important aspects that can reduce the risk of bias in an interventional animal experiment.

## Results and discussion

In the ARRIVE Guidelines, the results and discussion sections include “baseline data, numbers analyzed, outcomes and estimation, adverse events, interpretation/scientific implications, generalizability/translation, and funding”. The research in the present study showed that:

- ① Adverse events: The majority of animal experiments published in China (4278/4342, 98.5%) did not report details of important adverse events in experiments (item 17a). Researchers also need to pay attention to adverse events and analyze their nature, an important aspect for judging the pros and cons of an intervention. Therefore, accurate assessment and reporting of adverse events should not be neglected.
- ② Interpretation/scientific implications: Only 5.25% of the 4342 animal experiments published in Chinese journals reported limitations (item 18b) and only 0.35% described the significance of their research methods or findings in terms of the 3Rs principle (item 18c). The purpose of scientific design is to reduce any bias that may be experienced during experimentation. However, in terms of specific implementation, such as the animal model used, any potential source of bias in the experimental process, and factors affecting the accuracy of the study will reduce the scientific significance of the study [11]. Therefore, it is necessary for the author to objectively evaluate and explain the reasons in the discussion section of the article in order to ensure that the study remains scientifically valid while helping users understand the conclusions. In addition, the implementation of animal experiments should follow the 3Rs (replacement, refinement, and reduction of animals in research), the core of which is to protect, and use fewer, or zero, animals [1, 30]. Scientific experimental design can reduce the number of experimental animals [31, 32]. Experimental technology and optimized research procedures can reduce pain and anxiety in animals in experimental procedures [32]. Flecknell retrospectively studied the use and analgesic effects of perioperative and postoperative anesthesia in animal experimentation, suggesting that appropriate anesthesia and analgesic programs should be adopted in future animal studies and that animal welfare should be sufficiently considered beyond meeting legal requirements [33, 34]. Therefore, it is necessary for authors to interpret the scientific implications in the discussion section of the paper, which can help readers fully evaluate the research information, promote the implementation and the 3Rs principle, thereby improving the quality of the animal experiments.

There are a number of limitations to this study: ① it was based only on the evaluation of animal experimentation published in Chinese journals. The results may not represent the quality of the research of Chinese researchers published in foreign peer-reviewed journals; ② only interventional animal experiments were included. The results do not represent the quality of reports of other types of animal experiments.

## Conclusions

In summary, the “yes” compliance rate for Chinese animal experiments based on the 39 sub-items in the ARRIVE guidelines is not at a high level. In particular, insufficient reporting of methods, including an ethical statement, study design, experimental procedures, sample size, and allocation of animals to experimental groups provides evidence that researchers fail to fully understand the whole process of study design and implementation and, to a certain extent, hinder the transformation and utilization of research achievements. In addition, it should be pointed out that even though the criterion for achieving a “yes” classification in the present study was not particularly strict or rigorous, and the results of reporting quality remain rather poor. Additional problems would be exposed if studies were conducted in compliance with strict criteria. However, a randomized controlled trial conducted by Hair et al. [35] indicated that low compliance during the implementation of the ARRIVE guidelines may be due to the fact that journals only require authors to submit lists when they contribute, rather than editors having to review them according to the lists, hindering any improvement in the quality of experimental animal study reporting. Furthermore, we should also point out a number of existing problems in the current ARRIVE guidelines. Therefore, ARRIVE 2019 [36] has been optimized for rigor and transparency in comparison with the 2010 version, being more convenient for journals, institutions or researchers to understand and use by listing animal care and management separately. Therefore, the ARRIVE guidelines should be popularized, and researchers encouraged to follow them in the future, in particular, the ARRIVE guidelines should be promoted in specialized journals. Additionally, we also suggest that in practical research, animal ethics and welfare issues should be truly embraced. Where improvements to the design, implementation, and reporting of animal experiments are introduced, improvements in quality will ultimately follow.

## Methods

### Patient and public involvement

No patients or members of the public were involved in this study.

### Inclusion and exclusion criteria

#### Inclusion criteria

Interventional animal experiments with no restriction/limitation on animal species or type of intervention.

#### Exclusion criteria

Republished studies; Non-medical animal experiments; In vitro experiments based on animal tissues, organs or cells; Experiments involving both animals and humans; Relevant journals from Hong Kong, Macao, or Taiwan, which are limited by the authority.

### Search strategy

Searches of the Chinese Journal Full Text Database (CNKI), Chinese Academic Journal Database (Wanfang), Chinese Science and Technology Journal Database (VIP), and Chinese Biomedical Literature Database (CBM) from the inception of each database up to July 2018, were conducted. Search terms included: rats, rabbits, dogs, pigs, sheep, apes, frogs, orangutans, monkeys, animal experiments. A detailed search strategy for each database is presented in File S1.

### Methodological quality control

Relevant training was conducted for the researchers (B.Z., K.Y.H., Y.B.J., T.Z., Z.Z.S., W.Y.Z., F.C., F.M., Q.Q.G., and L.Z.) who participated in the project, including gaining an understanding and interpretation of the content of the checklist of the ARRIVE guidelines, evaluation principles and methods, and procedures and methods of literature screening and data extraction. After training, the researchers were tested on independent literature screening, data extraction, and quality evaluation prior to the start of the formal study, using 10% of the literature selected for the formal review. Consistency of the evaluation was evaluated. With a Kappa value > 0.8, consistency was strong, and the training considered qualified.

### Screened literature and extracted data

Four of the trained reviewers (B.Z., K.Y.H., Y.B.J., and T.Z.) independently screened, extracted and cross-checked studies based strictly on the inclusion and exclusion criteria. A third party (B.M.) judged any final decision in case of disagreement. A full-text data extraction table was created in advance, and completed during the extraction process, recording ① basic characteristics of the included studies: number of authors, the author’s names, dates, journal, animal species, sample size, intervention measures, etc.; ② Information for all items relating to the ARRIVE guidelines.

### Quality assessment of the included studies

Three reviewers (W.Y. Z., W.Y.Z., and F.C.) assessed the quality of the studies included in the review based on the 39 sub-items of 20 items included in the ARRIVE guidelines. We responded each item to “yes” (total or partial compliance) and “no” (noncompliance), where “yes” was defined as a study that reported all or part of at least one information item of a sub-item in detail. Conversely, a paper was “no” if it did not. For example, in Item 6 (Study design, Table 2), the item would only be classified as “yes” if at least one of the sub-items (6a-d) was reported, otherwise it would be classified as “no” (none of the 4 sub-items reported). Finally, the compliance rate of “yes” for the 39 sub-items was calculated, i.e. the total number of studies with “yes” relative to the total number of included studies.

### Subgroup analysis

Based on the year of publication (2010) of the ARRIVE guidelines, the included papers were analyzed comparatively (animal experiments published before 2010 vs. animal experiments published in 2010 or\ later). The “yes” compliance rate of 39 sub-items of animal experiments published before 2010 and those published in 2010 or later was calculated, respectively, i.e. the number of papers, of which each sub-item was assessed as “yes”, accounted for the percentage of the total number of animal experiments pre-ARRIVE and post-ARRIVE, respectively.

### Statistical analysis

Data were analyzed using SPSS software (v21.0, SPSS, Chicago, IL). Categorical data are presented as frequency (n) and percentage (%). A chi-square test was performed to compare percentages between groups. *P*-values < 0.05 were considered statistically significant.

## Supplementary Information

**Additional file 1: S1**. Chinese database search strategy.

## Data Availability

The datasets used and/or analysed during the current study are available from the corresponding author on reasonable request.
