# Supplementary material for: Quality of interventional animal experiments in Chinese journals: compliance with ARRIVE guidelines
Source: BMC Vet Res. 2020 Nov 26;16:460. doi: 10.1186/s12917-020-02664-1 (PMC7690085; doi:10.1186/s12917-020-02664-1)
Supplement: Supplementary file 1 — Additional file 1: S1. Chinese database search strategy. [file 12917_2020_2664_MOESM1_ESM.docx]

**附表：数据库检索策略**

| **1. China national knowledge infrastructure (CNKI) (Inception-July 2018): 1213**  **2. WanFang Data (Inception-July 2018): 5500** | | | | **3. China Science and Technology Journal Database (VIP)**  **(Inception-July 2018): 1702** | |
| --- | --- | --- | --- | --- | --- |
| #1 | 主题：兔 | | | #1 | 题名或关键字= 兔 |
| #2 | 主题：猪 | | | #2 | 题名或关键字= 猪 |
| #3 | 主题：羊 | | | #3 | 题名或关键字= 羊 |
| #4 | 主题：猩猩 | | | #4 | 题名或关键字= 猩猩 |
| #5 | 主题：鼠 | | | #5 | 题名或关键字= 鼠 |
| #6 | 主题：猴 | | | #6 | 题名或关键字= 猴 |
| #7 | 主题：狗 | | | #7 | 题名或关键字= 狗 |
| #8 | 主题：猿 | | | #8 | 题名或关键字= 猿 |
| #9 | 主题：蛙 | | | #9 | 题名或关键字= 蛙 |
| #10 | #1 OR #2 OR #3 OR #4 OR #5 OR #6 OR #7 OR #8 OR #9 | | | #10 | #1 OR #2 OR #3 OR #4 OR #5 OR #6 OR #7 OR #8 OR #9 |
| #11 | 主题：动物实验 | | | #11 | 题名或关键字= 动物实验 |
| #12 | #11 AND #12 | | | #12 | #11 AND #12 |
| **4. China Biology Medicine disc (CBM) (Inception-July 2018): 13298** | | | | | |
| #1 | 兔 | #13 | 鼠 | #25 | “蛙” [不加权:扩展] |
| #2 | “兔” [不加权:扩展] | #14 | “鼠” [不加权:扩展] | #26 | #24 OR #25 |
| #3 | #1 OR #2 | #15 | #13 OR #14 | #27 | #3 OR 6 OR #9 OR #12 OR #15 OR #18 OR #21 OR #23 OR 26 |
| #4 | 猪 | #16 | 猴 | #28 | 动物实验 |
| #5 | “猪” [不加权:扩展] | #17 | “猴” [不加权:扩展] | #29 | “动物实验” [不加权:扩展] |
| #6 | #4 OR #5 | #18 | #16 OR #17 | #30 | #28 OR #29 |
| #7 | 羊 | #19 | 狗 | #31 | #27 AND # 30 |
| #8 | “羊” [不加权:扩展] | #20 | “狗” [不加权:扩展] |  |  |
| #9 | #7 OR #8 | #21 | #19 OR #20 |  |  |
| #10 | 猩猩 | #22 | 猿 |  |  |
| #11 | “猩猩” [不加权:扩展] | #23 | “猿” [不加权:扩展] |  |  |
| #12 | #10 OR #11 | #24 | 蛙 |  |  |

[**Attached**](javascript:;) [**list**](javascript:;)**: databases search strategy**

| **1. China national knowledge infrastructure (CNKI) (Inception-July 2018): 1213**  **2. WanFang Data (Inception-July 2018): 5500** | | | | **3. China Science and Technology Journal Database (VIP)**  **(Inception-July 2018): 1702** | |
| --- | --- | --- | --- | --- | --- |
| #1 | Subject: rabbits | | | #1 | title/abstract = rabbits |
| #2 | Subject: pigs | | | #2 | title/abstract = pigs |
| #3 | Subject: sheep | | | #3 | title/abstract = sheep |
| #4 | Subject: orangutans | | | #4 | title/abstract = orangutans |
| #5 | Subject: rats | | | #5 | title/abstract = rats |
| #6 | Subject: monkeys | | | #6 | title/abstract = monkeys |
| #7 | Subject: dogs | | | #7 | title/abstract = dogs |
| #8 | Subject: apes | | | #8 | title/abstract = apes |
| #9 | Subject: frogs | | | #9 | title/abstract = frogs |
| #10 | #1 OR #2 OR #3 OR #4 OR #5 OR #6 OR #7 OR #8 OR #9 | | | #10 | #1 OR #2 OR #3 OR #4 OR #5 OR #6 OR #7 OR #8 OR #9 |
| #11 | Subject: animal experiments | | | #11 | title/abstract = animal experiments |
| #12 | #11 AND #12 | | | #12 | #11 AND #12 |
| **4. China Biology Medicine disc (CBM) (Inception-July 2018): 13298** | | | | | |
| #1 | Rabbits | #12 | #10 OR #11 | #23 | “apes”[unweighted : [extension](javascript:;)] |
| #2 | “rabbits”[unweighted: [extension](javascript:;)] | #13 | Rats | #24 | Frogs |
| #3 | #1 OR #2 | #14 | “rats”[unweighted: [extension](javascript:;)] | #25 | “frogs” [ unweighted: [extension](javascript:;)] |
| #4 | Pigs | #15 | #13 OR #14 | #26 | #24 OR #25 |
| #5 | “pigs” [unweighted: [extension](javascript:;)] | #16 | Monkeys | #27 | #3 OR 6 OR #9 OR #12 OR #15 OR #18 OR #21 OR #23 OR 26 |
| #6 | #4 OR #5 | #17 | “monkeys”[unweighted: [extension](javascript:;)] | #28 | Animal experiments |
| #7 | Sheep | #18 | #16 OR #17 | #29 | “animal experiments” [ unweighted: [extension](javascript:;)] |
| #8 | “sheep” [unweighted: [extension](javascript:;)] | #19 | Dogs | #30 | #28 OR #29 |
| #9 | #7 OR #8 | #20 | “dogs”[unweighted: [extension](javascript:;)] | #31 | #27 AND # 30 |
| #10 | orangutans | #21 | #19 OR #20 |  |  |
| #11 | “orangutans”[unweighted: [extension](javascript:;)] | #22 | Apes |  |  |
